# Primary care physicians comprehensively manage acute pulmonary embolism without higher-level-of-care transfer: A report of two cases

**DOI:** 10.1097/MD.0000000000023031

**Published:** 2020-11-06

**Authors:** Dayna J. Isaacs, Elizabeth J. Johnson, Erik R. Hofmann, Suresh Rangarajan, David R. Vinson

**Affiliations:** a University of California, Davis, School of Medicine; b Department of Emergency Medicine, UC Davis Health, Sacramento; c The Permanente Medical Group, Oakland; d Department of Emergency Medicine, Kaiser Permanente South Sacramento Medical Center, Sacramento; e Department of Adult and Family Medicine, Kaiser Permanente Oakland Medical Center, Oakland; f Department of Emergency Medicine, Kaiser Permanente Roseville Medical Center, Roseville; g Kaiser Permanente Northern California Division of Research, Oakland, CA.

**Keywords:** case report, outpatient management, primary care, pulmonary embolism, risk stratification

## Abstract

**Rationale:**

The evidence for outpatient pulmonary embolism (PE) management apart from hospitalization is expanding. The availability and ease of direct oral anticoagulants have facilitated this transition. The literature, however, is sparse on the topic of comprehensive management of pulmonary embolism in the primary care clinic setting. As such, the role of the primary care physician in the complete diagnosis, risk stratification for outpatient eligibility, and initiation of treatment is unclear.

**Case presentations:**

Case 1: A 33-year-old man with known heterozygous Factor V Leiden mutation and a remote history of deep vein thrombosis presented to his primary care physician's office with 2 days of mild pleuritic chest pain and a dry cough after a recent transcontinental flight. Case 2: A 48-year-old man with a complex medical history including recent transverse myelitis presented to his primary care family physician with dyspnea and pleuritic chest pain for 6 days.

**Diagnosis:**

Case 1: Computed tomographic pulmonary angiography that same afternoon showed multiple bilateral segmental and subsegmental emboli as well as several small pulmonary infarcts. Case 2: The patient's D-dimer was elevated at 1148 ng/mL. His physician ordered a computed tomographic pulmonary angiography, performed that evening, which showed segmental and subsegmental PE.

**Interventions:**

Both patients were contacted by their respective physicians shortly after their diagnoses and, in shared decision-making, opted for treatment at home with 5 days of enoxaparin followed by dabigatran.

**Outcomes:**

Neither patient developed recurrence nor complications in the subsequent 3 months.

**Lessons:**

These cases, stratified as low risk using the American College of Chest Physicians criteria and the PE Severity Index, are among the first in the literature to illustrate comprehensive primary care-based outpatient PE management. Care was provided within an integrated delivery system with ready, timely access to laboratory, advanced radiology, and allied health services. This report sets the stage for investigating the public health implications of comprehensive primary care-based PE management, including cost-savings as well as enhanced patient follow-up and patient satisfaction.

## 1 Introduction

The evidence for the outpatient management of select ambulatory patients with pulmonary embolism (PE) is expanding.^[1–3]^ In reducing hospitalizations, this shift to outpatient care is expected to reduce healthcare costs and improve patient quality of life. The role of the primary care physician or general practitioner in this expansion, however, is unclear.^[4,5]^ The literature describes their part only in determining which patients with possible PE should be referred for diagnostic imaging or definitive management.^[6–9]^ Transfer of the patient with probable PE to a higher level of care has long been the convention. It is typically the receiving specialist or hospital-based emergency physician who identifies which patients with acute PE are eligible for ambulatory care, initiates anticoagulation, and arranges home discharge and close follow-up.^[10,11]^

Yet resources are at hand for the primary care physician to continue outpatient PE management beyond the initial diagnostic evaluation without having to transfer every patient with possible PE to a higher level of care. Validated risk stratification tools are now readily available to help identify patients with acute PE who can be safely managed without hospitalization.^[12]^ Treatment of PE has also been simplified, thanks to the advent of direct (or non-vitamin K) oral anticoagulants, which may obviate the need for injectable medications. In many practice settings, thrombosis specialists can be consulted remotely for expert opinion and management advice. Comprehensive primary care-based outpatient PE management is beginning to emerge in some care delivery systems, but descriptions of such practices in the literature are uncommon.^[4,5]^ So, what does exclusive primary care-based PE management look like? We describe 2 cases treated in an integrated delivery system, which may be particularly well-suited for this novel approach.

The patients provided written informed consent. Approval by our institutional review board is not required for small case reports. This research was conducted according to the principles of the Declaration of Helsinki.

## 2 Case presentations

### 2.1 Case 1

This 33-year-old man had known heterozygous Factor V Leiden mutation and a remote history of deep vein thrombosis following international travel. He was no longer on anticoagulation. He had flown back to California from Europe 10 days earlier. He presented to his primary care internist with 2 days of mild pleuritic chest pain and a dry cough. He denied shortness of breath and lower extremity complaints. His vital signs were: systolic blood pressure 115 mm Hg, pulse 95, temperature 99.0°F, and oxygen saturation 98%. His heart had a regular rate and rhythm, his lungs were clear to auscultation bilaterally, and his lower extremities showed no signs of deep vein thrombosis. The remainder of his physical examination was normal. Both a 12-lead electrocardiogram and a 2-view chest radiograph were normal. Computed tomographic pulmonary angiography that same afternoon showed multiple bilateral segmental and subsegmental emboli as well as several small pulmonary infarcts. He returned to his physician's office that same day to discuss the results. Through shared decision-making, they opted to treat at home with 5 days of enoxaparin followed by dabigatran, the preferred PE treatment regimen in the medical group at the time. The next day he had his first telephone consultation with pharmacy-led Anticoagulation Management Services and the day thereafter a secure email exchange with his physician. A hematologist was consulted for long-term management recommendations. At 6 months, the patient had discontinued anticoagulation as planned, except for pre-flight prophylaxis. He had no complications, nor recurrence 3 months after stopping treatment.

### 2.2 Case 2

This 48-year-old man had a history of hypertension, coronary artery disease, and transverse myelitis diagnosed 2 months prior for which he was taking daily prednisone. He had no history of venous thromboembolic disease. He presented to his primary care family physician for dyspnea and pleuritic chest pain for 6 days. He denied cough, hemoptysis, fever, lower extremity complaints, and other recent illness. His vital signs were: systolic blood pressure 115 mm Hg, pulse 92, temperature 97.7°F, and oxygen saturation 97%. His heart had a regular rate and rhythm, his lungs were clear to auscultation bilaterally, and his lower extremities showed no signs of deep vein thrombosis. The remainder of his exam was normal. He had an unremarkable 12-lead electrocardiogram and 2-view chest radiograph. His B-type natriuretic peptide level was normal, but his D-dimer was elevated at 1148 ng/mL (normal is < 500 ng/mL). His physician ordered computed tomographic pulmonary angiography, performed that evening, which showed segmental and subsegmental PE. The results were communicated the next morning to a covering family physician, who called the patient to discuss his diagnosis and treatment plan. They opted for outpatient management, involving 5 days of enoxaparin followed by dabigatran. Anticoagulation Management Services also contacted the patient that day for further education and follow-up. He developed no recurrence or complications in the subsequent 3 months and has continued with long-term anticoagulation.

## 3 Discussion

This report is among the first in the literature to describe a new outpatient venue of comprehensive PE care delivery for stable ambulatory patients—the primary care or general practice clinic setting.^[4,5]^ These cases illustrate successful adoption of practices historically assigned to the emergency department or specialty clinic. At the core of this transformation of care is proper patient selection. Both of these patients met the American College of Chest Physicians criteria for outpatient care: “clinically stable with good cardiopulmonary reserve; no contraindications such as recent bleeding, severe renal or liver disease, or severe thrombocytopenia (i.e., < 70,000/mm^3^); expected to be compliant with treatment; and the patient feels well enough to be treated at home.”^[1]^ These patients had no comorbid conditions (cancer, heart failure, or chronic lung disease) or abnormal physical examination findings (e.g., tachycardia ≥110 beats/min) found in the PE Severity Index to be associated with 30-day all-cause mortality. They were classified as low risk on the PE Severity Index (Classes I-II), and, therefore, were potential candidates for outpatient care.^[13]^ These patients also lacked relative contraindications to ambulatory care used in the Canadian, Hestia, and Kaiser Permanente PE studies^[10,11,14,15]^ and had no contraindications to direct oral anticoagulants.

The physicians were able to diagnose PE because of ready, timely access to laboratory and advanced radiology services; diagnostic assessment was completed and treatment was initiated within 24 hours of the initial evaluation. Clinics that are unable to secure same-day or next-day computed tomography appointments may have no choice but to transfer care of stable ambulatory patients with an elevated D-dimer or a high pre-test probability of PE to a hospital-based ED or specialty clinic for advanced imaging. Another resource that facilitates comprehensive primary care-based PE management is the availability of a thrombosis expert to assist as needed with decision-making around patient eligibility, medication selection, and duration of treatment. Such consultation is particularly germane for physicians unaccustomed to providing comprehensive care but required to do so when their newly diagnosed PE patient declines transfer to the emergency department, as may happen on occasion.^[5]^ Note that these 2 patients received close follow-up, both by their primary care physicians and Anticoagulation Management Services,^[16]^ though the latter is not essential for comprehensive primary care-based PE management.

In the 30 days following their initial diagnosis, the patients did not require emergency department or inpatient care for PE-related complaints, nor suffered bleeding, recurrent thromboembolism, or death. The combination of low PE Severity Index scores and favorable short-term outcomes suggests that careful patient selection was at work.

These patients were asked the following question at the time of securing consent for this report: “Dr. [Name] informed you that your scan of the chest had revealed a blood clot in the lungs, and that you could pick up your medication for treatment at home. At that point, would you have preferred to have gone to the emergency department for re-evaluation, treatment, and possible overnight observation?” Both patients answered in the negative.

The physicians who cared for these patients practiced in 2 different outpatient clinics in northern California, both located on or near their respective medical centers, giving them ready access to advanced imaging services. One primary care physician cared for their own patient from start to finish, while another shared their patient's care with a colleague during cross-coverage. The physicians were residency-trained and board-certified in family medicine or internal medicine.

## 4 Conclusion

This report introduces comprehensive primary care-based PE management to the literature as a novel, feasible approach for select low-risk ambulatory patients without having to transfer care to the emergency department, specialty clinic, or inpatient setting. This exclusive primary care clinic-based approach may be a safe and effective option with an eligible low-risk patient, a knowledgeable physician with an accommodating schedule, and a supportive practice setting that has ready access to diagnostic testing, advanced imaging, and close follow-up.^[4,5]^ It is unknown which low-risk patients are best suited for this model of care and how it compares with conventional emergency department transfer regarding clinical outcomes. When done safely, we anticipate that comprehensive primary care-based PE management will correlate with decreased healthcare costs, improved patient follow-up, and enhanced patient satisfaction.

## Acknowledgments

We are grateful for the support of the Kaiser Permanente Northern California's Community Benefit Program and The Permanente Medical Group's Delivery Science and Physician Researcher Programs. We are grateful as well to our two patients for their participation in this report.

## Author contributions

**Conceptualization:** Dayna J Isaacs, Elizabeth J Johnson, Erik R Hofmann, Suresh Rangarajan, David R Vinson.

**Data curation:** Dayna J Isaacs, Elizabeth J Johnson, Erik R Hofmann, Suresh Rangarajan, David R Vinson.

**Project administration:** David R Vinson.

**Supervision:** David R Vinson.

**Writing – original draft:** David R Vinson.

**Writing – review & editing:** Dayna J Isaacs, Elizabeth J Johnson, Erik R Hofmann, Suresh Rangarajan, David R Vinson.
